# Distinct predictive performance of Rac1 and Cdc42 in cell migration

**DOI:** 10.1038/srep17527

**Published:** 2015-12-04

**Authors:** Masataka Yamao, Honda Naoki, Katsuyuki Kunida, Kazuhiro Aoki, Michiyuki Matsuda, Shin Ishii

**Affiliations:** 1 Graduate School of Informatics, Kyoto University, Sakyo, Kyoto, Japan; 2 Imaging Platform for Spatio-temporal Information, Graduate School of Medicine, Kyoto University, Sakyo, Kyoto, Japan; 3 Graduate School of Science, University of Tokyo, Bunkyo, Tokyo, Japan; 4 Graduate School of Biostudies, Kyoto University, Sakyo, Kyoto, Japan

## Abstract

We propose a new computation-based approach for elucidating how signaling molecules are decoded in cell migration. In this approach, we performed FRET time-lapse imaging of Rac1 and Cdc42, members of Rho GTPases which are responsible for cell motility, and quantitatively identified the response functions that describe the conversion from the molecular activities to the morphological changes. Based on the identified response functions, we clarified the profiles of how the morphology spatiotemporally changes in response to local and transient activation of Rac1 and Cdc42, and found that Rac1 and Cdc42 activation triggers laterally propagating membrane protrusion. The response functions were also endowed with property of differentiator, which is beneficial for maintaining sensitivity under adaptation to the mean level of input. Using the response function, we could predict the morphological change from molecular activity, and its predictive performance provides a new quantitative measure of how much the Rho GTPases participate in the cell migration. Interestingly, we discovered distinct predictive performance of Rac1 and Cdc42 depending on the migration modes, indicating that Rac1 and Cdc42 contribute to persistent and random migration, respectively. Thus, our proposed predictive approach enabled us to uncover the hidden information processing rules of Rho GTPases in the cell migration.

Living cells process extracellular and intracellular information employing biochemical reaction network that we call “signal transduction”. Great deal of molecular components in signal transduction has been extensively identified. However, the intracellular information processing remains poorly understood. Over the past decade, live-cell imaging techniques have been developed to visualize the dynamics of molecular activity *in situ* by means of biosensors, for example, based on the principle of Förster (or fluorescence) resonance energy transfer (FRET)^1^,^2^,^3^. Thus, we are entering a new era to investigate a question of how molecular signals are dynamically processed through signaling cascade. To examine this issue, we focused on cell migration as a model biological system, because both molecular activity (as input signal) and morphological changes (as output signal) during cell migration can be monitored by FRET imaging.

Cell migration plays important roles in various biological functions, including wound healing, embryonic development, and cancer invasion^4^. This process is highly complex and coordinated in space and time; the protrusion and retraction of cellular membranes are primarily driven by the cytoskeleton, the reorganization of which is regulated by intracellular signaling^5^,^6^. Many studies have extensively investigated the molecular mechanisms involved in cell migration and have recognized the Rho small GTPases as key regulators of actin dynamics^7^,^8^. Rac1 and Cdc42 were classically thought to induce lamellipodia and filopodia, respectively^7^,^9^, and their downstream pathways have been well identified. For example, Rac1 activates WAVE complex and Arpin that respectively up- and down-regulates Arp2/3 complex to induce branched network of F-actin in lamellipodia^10^, whereas Cdc42 activates actin-associated proteins, including fascin, formin (mDia2) and Ena/VASP, to induce F-actin bundles in filopodia^11^,^12^. In addition, Rac1 and Cdc42 overlappingly activate the same collections of proteins to regulate F-actin, microtubules, adhesion and actomyosin assembly^13^. In spite of such accumulating knowledge of Rac1 and Cdc42 downstream pathways, little is known about the functional differences between Rac1 and Cdc42, in particular, how these Rho GTPases participate in cell migration. Moreover, a quantified discussion has been missing: although these molecules are involved in cell migration, how much are they quantitatively responsible for? 

Based on these FRET imaging techniques, we previously examined the relationship between the activities of Rho GTPases and morphological changes, both of which were quantified using automated image-processing techniques^14^,^15^. In those studies, our cross-correlation analysis revealed that local membrane elongation preceded the Rac1 and Cdc42 activation by 30–60 seconds, which was then found to be consistent with another study^16^. This finding was counterintuitive to the common idea that the intracellular Rho GTPases regulate morphological changes via cytoskeletal reorganization^17^,^18^. Related questions have arisen: Do the activities of Rac1 and Cdc42 truly cause cellular morphodynamics and migration ? If they are the putative causes, how and how much do these molecules regulate morphodynamics and migration?

A cross-correlation analysis is not suitable to answer these questions because it only describes a one-to-one relationship between the molecular activity at time *t* and morphological change at another time *t’*^14^,^15^,^16^. There may be a causal relationship in which the molecular activity at time *t* has sustained, rather than instantaneous, effects on future morphological changes. In other words, the morphological change could be determined by the history of molecular activities. Under such an assumption, a multi-to-one relationship between the time-series of molecular activities until *t* and morphological change at *t* should be examined. A solution to this end, developed in the field of control theory, is to estimate a response function^19^,^20^,^21^, which is defined as a spatiotemporal response of morphological changes to instantaneous and local molecular activation.

What is the physiological meaning of the response function of Rho GTPases ? The response function can be seen as representing intracellular information processing as a whole, as opposed to identifying the detailed pathway itself. For example, if the Rho GTPase signal is transmitted to a downstream pathway with incoherent feedforward loop in which Rho GTPase rapidly and slowly up-regulates activators and inhibitors for the morphological change, respectively^22^, the response function turns from positive to negative as time proceeds. Like this, the profile of the response function is determined by kinetics of the downstream pathway. Therefore, identification of the response function profile is physiologically important for understanding downstream kinetics-based information processing.

A challenging task is to identify the response function based on time-lapse FRET imaging data, which goes beyond a simple correlation analysis^23^,^24^. A significant correlation between two cellular factors does not mean a significant causality between them, because they could both have a common cause. Identifying the response function can yield meaningful knowledge of whether molecular activity has a potential causal link to predict morphological changes using the response function^23^,^24^. Such predictive performance provides a new quantitative measure of how much the Rho GTPases contribute to the cell migration.

In this study, to elucidate and quantify the hidden information processing rules of intracellular signaling in cellular morphodynamics and migration with the help of FRET live-cell imaging, we proposed a new decoding-based predictive approach in combination with image processing and statistical signal processing.

## Results

**Figure 1:**
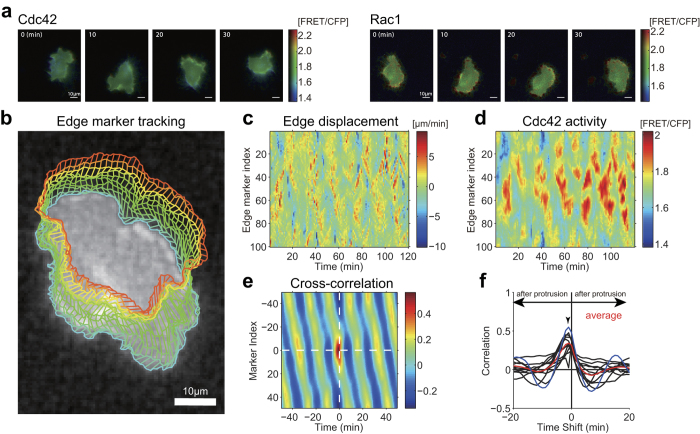
Quantification of cell edge displacement and Rho GTPase activity. **(a)** Snapshot images showing the spontaneous migration of HT-1080 cells expressing the biosensors Raichu–Cdc42 (left) and Raichu–Rac1 (right). See also Supplementary Movies 1,2. FRET/CFP ratio ranges in intensity modulated display (IMD) mode, which associates color hue with emission ratio value and the intensity of each hue with the source image brightness, are shown at the right of each image. (**b**) Migrating HT1080 cells are tracked based on the CFP image. Different-colored polygons (connected markers) indicate the time-series of the tracked cellular boundaries (from cyan to red). The vertices (virtual markers) are placed in an equidistant manner along the perimeter at each time. Edge displacement is quantified based on the length of the connected link between the vertices at serial time frames. (**c,d**) Quantified edge displacement (elongation/retraction) (**b**) and Cdc42 activity, i.e., the FRET/CFP ratio value **(c)** at each virtual marker is mapped onto a two-dimensional heat map consisting of time (abscissa) and marker index (ordinate). (**e**) The spatiotemporal cross-correlation function between the edge displacement and Cdc42 activity of a specific cell (the same one as in (**c,d**)) is plotted. Abscissa and ordinate indicate the shifts in time and marker indices, respectively. (**f**) The temporal cross-correlation functions between edge displacement and Cdc42 activity are plotted with the time shift. The blue line was calculated by (**c**) and **(d)**. In each sample (a single black line), the local shape change precedes the molecular activity change. The red line shows the mean cross-correlation function of all of the cells (N = 11).

Using image processing and a statistical decoding technique, this study aimed to identify the processing mechanism by which Rac1 and Cdc42 control cellular morphodynamics and migration. The activities of Rac1 and Cdc42 were monitored using FRET time-lapse imaging in human HT-1080 fibrosarcoma cells as a model system of spontaneous random migration (Fig. 1a; Supplementary Movies 1–2)^1^,^25^.

### Quantification of Rho GTPase Activity and Edge Displacement

We developed an improved image-processing algorithm to more robustly quantify local displacements, i.e., the elongation and retraction of the lamellipodia, compared with previous algorithms^14^,^16^. Note that HT-1080 cells exhibited no or very few clear filopodial structures in our culture condition. First, virtual markers were placed on the cellular periphery in an equidistant manner to track the morphological changes during cell migration. These virtual markers tracked the local displacements of the cellular edge such that the sum of the travelling distances of the markers was minimized with the constraint that the adjacent markers remained equidistant (see Materials and Methods). Using this method, we were able to stably quantify the level of protrusion/retraction in the local membrane for an extended period (Fig. 1b). Next, the local morphological changes were represented in a heat map with the abscissa and ordinate mapped to the time and peripheral position, respectively (Fig. 1c and Supplementary Fig. S1a). We also quantified the activity levels of the Rac1 and Cdc42 near the virtual markers (Fig. 1d and Supplementary Fig. S1b) (see Materials and Methods). Because these heat maps shared the common coordinates of time and peripheral position, we could consistently calculate a spatiotemporal correlation between the molecular activity levels and the local morphological changes (Fig. 1e and Supplementary Fig. S1c), which clearly showed the same characteristics as previously reported; namely, protrusion (retraction) preceded the activation (inactivation) of Rac1 and Cdc42^14^,^15^,^26^. These characteristics were fairly well conserved among different HT1080 cells (11 cells for Cdc42 and 10 cells for Rac1) (Fig. 1f and Supplementary Fig. S1d). These results raised a further question: If the morphological changes precede the changes in Rac1 and Cdc42 activity, how do these two Rho GTPases regulate cellular morphodynamics and migration ? 

### Prediction of Edge Evolution Using Rho GTPase Activity

To answer this question, we applied a decoding technique to identify the response functions of the Rho GTPases for morphological change. We constructed a regression model in which the local morphological change of a specific time frame is given using a weighted-summation of the past and nearby molecular activities:


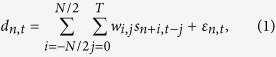


where *d*_*n*,*t*_ and *s*_*n*,*t*_ denote the displacement of the cellular edge and molecular activity of either Cdc42 or Rac1, respectively, on the *n*-th virtual marker at time *t, w*_*i,j*_ is a regression parameter for the spatial shift *i* and delay time *j*, and *ε*_*n,t*_ is white Gaussian noise*. d*_*n*,*t*_ and *s*_*n*,*t*_ are values of the (*n*,*t*)-th elements of the two heat maps in Fig. 1b,c, respectively. Given that the effect of the molecular activity spatially propagates along the cellular edge in a symmetric manner, we applied a spatially symmetric character to the regression parameters: *w*_*k,j*_ =_* *_*w*_*−k,j*_. We call the set of regression parameters *w*_*i.j*_ (*i* = −*N*/2, …, *N*/2; *j* = 0, …, *T*) a response function, where *T* and *N* are the maximum delay time and the number of virtual markers (i.e., the resolution level of the entire cellular periphery), respectively. Note that *n* is on a circular coordinate: *d*_*n + N*,*t*_ = *d*_*n*,*t*_ and *s*_*n + N*,*t*_ = *s*_*n*,*t*_. This regression model has a property that if local and impulse molecular activation *s*_*n,t*_ = *δ*_*n,0*_*δ*_*t,0*_ (*δ*_*i,j*_ is a Kronecker’s delta function) is applied, the induced spatiotemporal response of edge displacement can be simply described by *w*_*n,t*_.

**Figure 2:**
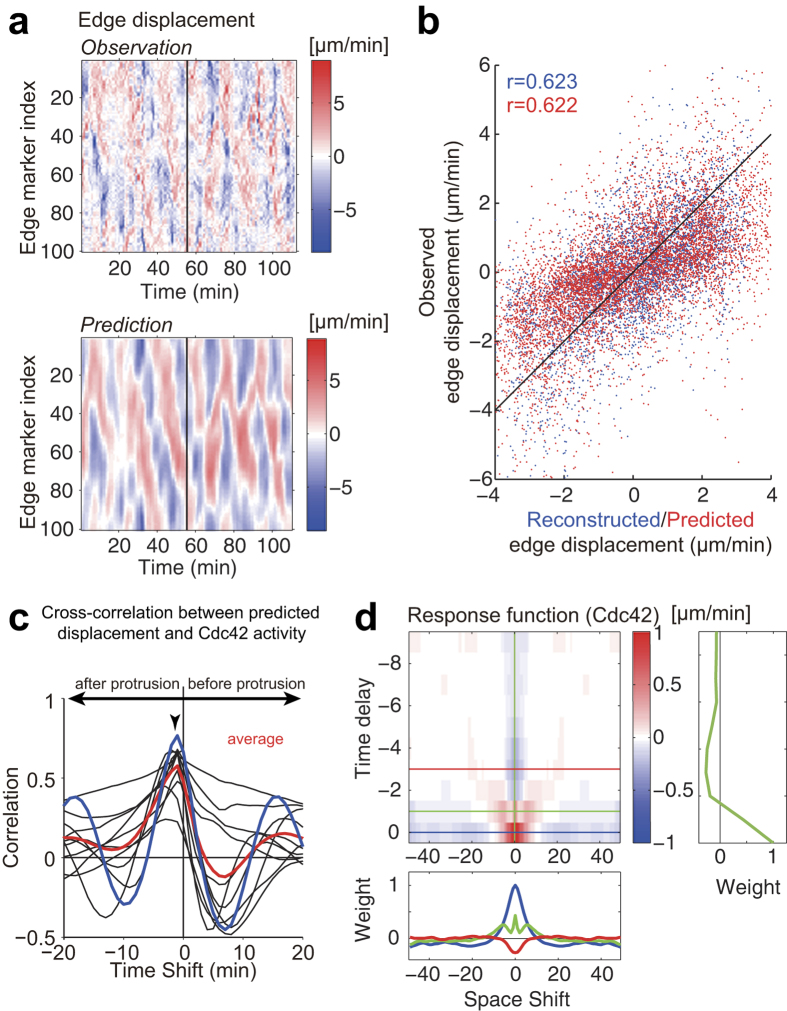
Prediction of elongation/retraction based on Cdc42 activity via the response function. **(a)** Prediction of the local morphological change based on Cdc42 activity. The upper and lower panels show the local edge displacement (same as Fig. 1c) obtained in an experiment and the local edge displacement reconstructed/predicted via the response function, respectively. The left-hand side of the vertical black line in the upper panel was used as the training data to estimate the response function, and hence the right-hand side was never used for the training. For validation, the left-hand and right-hand sides in the lower panel were reconstructed and predicted using the estimated response function, respectively. (**b**) The reconstructed and predicted edge displacements were strongly correlated with the observed displacements. Each dot represents the relationship between the reconstruction/prediction and an observation of each edge displacement, and the red and blue colors correspond to reconstruction and prediction, respectively. (**c**) The temporal cross-correlation functions between the predicted edge displacement and the Cdc42 activity are plotted with the time shift as in Fig. 1f. (**d**) A response function of Cdc42 is plotted on a two-dimensional plane (upper left panel) coordinated by a space shift and a time delay. Each colored-line in the upper right and lower panels represents a cross-section of the spatiotemporal response function (upper left panel) along the straight line with the corresponding color.

Next, we estimated the response function to fit the first half of the experimental time series dataset, called a training dataset, by applying a ridge regression technique, which prefers a smooth response function (see Materials and Methods). The estimated response function was then validated not only by reconstructing the local edge displacement in the training dataset but also by predicting the local edge displacement in the latter half of the time series, which is called a validation dataset (Fig. 2a,b for Cdc42; Supplementary Fig. S2a,b for Rac1). Statistical tests showed that our response function-based prediction was significantly better than the simple prediction that did not use the history of molecular activities (see Materials and Methods). In addition, we found that the predicted membrane protrusion (retraction) preceded the molecular activation (inactivation) (Fig. 2c for Cdc42; Supplementary Fig. S2c for Rac1), similarly to the prior counterintuitive observation^14^,^15^,^26^ (Fig. 1f for Cdc42; Supplementary Fig. S1d for Rac1).

The identified response functions of Rac1 and Cdc42 are represented in the time-space domain (Fig. 2d for Cdc42; Supplementary Fig. S2d for Rac1). We found that Rac1 and Cdc42 shared similar profiles of the response functions; the response function at the origin (*i* = 0, *j* = 0) takes a large positive value but becomes negative as the time and position deviate from the origin, suggesting that the Rac1 and Cdc42 activities transiently and locally induce membrane protrusion and that the local protrusion is accompanied by global retraction of the remaining region, possibly due to mass conservation of the cell volume. As the time is prolonged, moreover, the response function takes on a characteristic V-shape, suggesting that the membrane protrusion induced by Rac1 and Cdc42 propagates laterally. We also noticed that the response function was endowed with the property of a differentiator; the temporal aspect of the response function changed from a large positive value to a negative value as time progressed (right panel in Fig. 2d for Cdc42; Supplementary Fig. S2d for Rac1), indicating that the membrane protrusion was induced by a temporal derivative of the Rac1 and Cdc42 activities, in particular at the initial phase of the activity increase. We suggest that the aforementioned counterintuitive observation is a side effect caused by the downstream signaling of the Rho GTPases working as temporal differentiators (see Discussion).

### Inter-cellular Analysis

**Figure 3:**
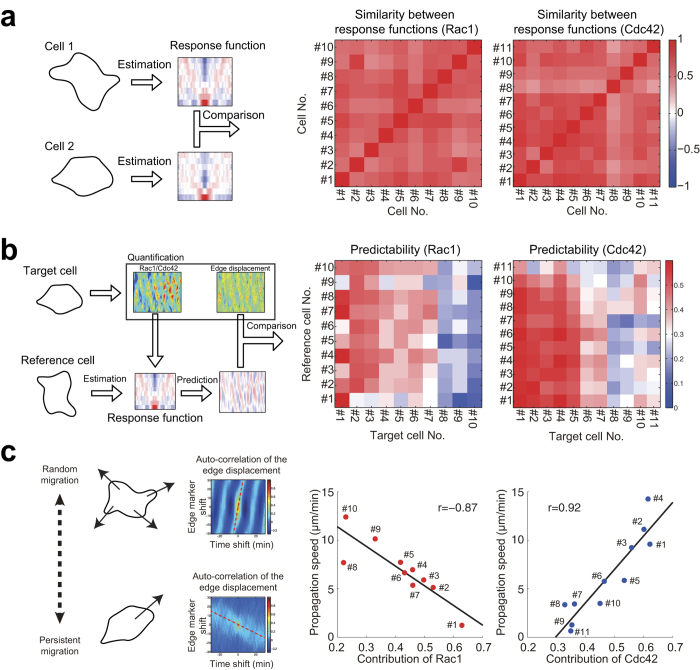
Cross-cellular analysis of response functions. **(a)** Schematic of the similarity analysis of the response functions of Rac1 (middle panel) and Cdc42 (right panel) between different cells. Both the ordinate and abscissa indicate indices of cells so that each matrix element represents the cosine similarity in the response functions between the pair of cells specified by the element’s indices. (**b**) Predictability of response functions of Rac1 (middle panel) and Cdc42 (right panel) between different cells. Each matrix element represents the correlation between the observed edge displacement in a specific cell (‘target cell’) and the predicted edge displacement in the target cell decoded based on the response function of another cell (‘reference cell’). The ordinate and abscissa denote the indices of the target cell and the reference cell, respectively. (**c**) Relationship between the mode of the cell migration and the contributions of Rac1 (middle panel) and Cdc42 (right panel) to the morphological change. The contributions of Rac1 and Cdc42 were evaluated via predictability, which corresponds to the diagonal elements in (**b**). Each dot represents a single cell, where cell numbers with blue and red dots correspond to those in (**b**), respectively. The migration mode was evaluated via the lateral propagation speed of the edge displacement, which was quantified based on the speed of the wave propagation, which is indicated with a red dashed line in the spatiotemporal auto-correlation function (left insets).

To validate the consistency of our approach, we examined the similarity of the response functions estimated from different cells, i.e., a cross-cellular analysis of the response functions (see Materials and Methods) (Fig. 3a). The similarity indices exhibited high values across the cells, indicating that essentially the same decoding machinery with the V-shaped profiles of the response function transformed Rac1 and Cdc42 activities into cellular morphologies. We furthermore validated cross-cellular predictability of the response functions (Fig. 3b); i.e., the morphological changes of a certain cell (‘target cell’) were decoded based on the response function estimated from another cell (‘reference cell’). Interestingly, we found two clusters in terms of cross-cellular predictability (Fig. 3b): ‘predictable cells’ and ‘unpredictable cells’ (cell indices from 1 to 7 and from 8 to 10 in middle panel for Rac1, respectively; cell indices from 1 to 7 and from 8 to 11 in right panel for Cdc42, respectively). The variance in the prediction error was significantly smaller than that of the molecular activity in the predictable cells (*p* < 0.01 for all of the predictable cells on Cdc42 imaging, and *p* < 0.01 for all of the predictable cells on Rac1 imaging according to the *F*-test). However, the variance in the prediction error was not significantly different from that of the edge displacement in part of the unpredictable cells.

Accordingly, the morphological changes in the predictable cells were reliably decoded via other cells’ response functions, whereas the changes in the unpredictable cells were minimally decoded not only via other cells’ response functions but also via their own response functions (Fig. 3b) (diagonal components indexed from 6 to 11 in middle panel for Rac1; diagonal components indexed from 8 to 10 in right panel for Cdc42). These results, accompanied by the finding that the response functions were consistent across different cells (Fig. 3a), imply that our statistical analysis reliably captured characteristic response functions even for unpredictable cells and also that the morphological changes of the unpredictable cells may be perturbed by another pathway.

### Contribution of Rac1 and Cdc42 to the Cell Migration

What is the primary difference between ‘predictable cells’ and ‘unpredictable cells’? The pattern of cellular morphodynamics could be a key to answering this question. To identify a pattern in the morphological changes, we calculated the spatiotemporal auto-correlation (auto-correlation map) of the edge displacements for each cell (see Materials and Methods) (left two insets in Fig. 3c). We found two patterns of morphological changes; in the cells migrating randomly in a back-and-forth manner, the local protrusion spatially propagated laterally in a wave-like pattern, whereas in the cells persistently migrating in a single direction, the local protrusion remained within a restricted region. When the propagation speed of the local protrusion was estimated from the auto-correlation map, we found that the predictabilities of the morphological changes decoded via the response functions of Rac1 and Cdc42 were negatively and positively correlated with the propagation speed, respectively (Fig. 3c). This result also suggests that the predictability could be interpreted as contributions of Rac1 and Cdc42 to cell migration. Taken together, a balance between the contributions of Rac1 and Cdc42 could determine the mode of migration.

### Predicting the Macroscopic Behavior of the Migrating Cells

**Figure 4:**
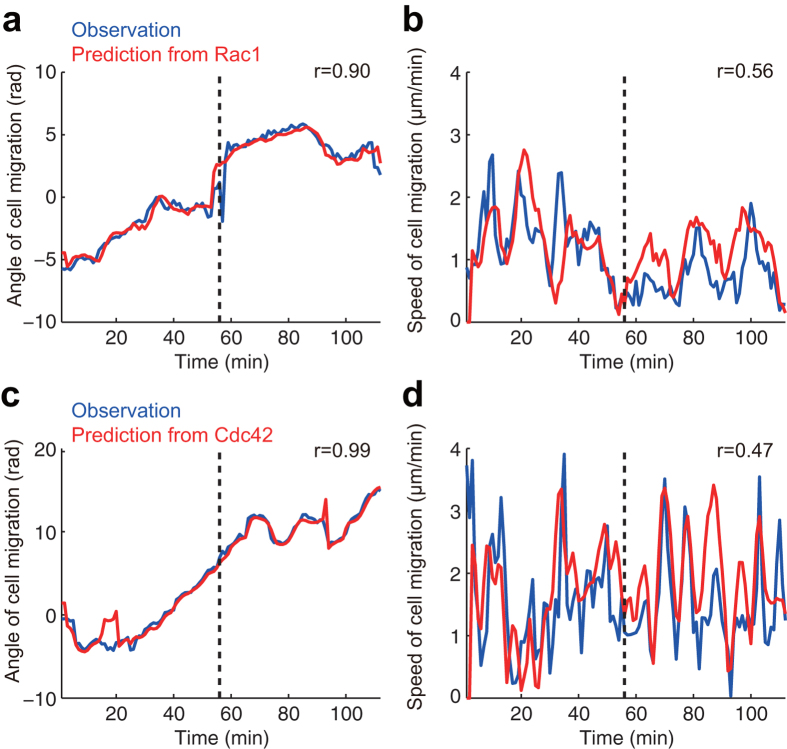
Predictions of macroscopic cell migration based on Cdc42 activity. Macroscopic cell migration is reconstructed/predicted from Rac1 (**a,b**) and Cdc42 (**c,d**) activity. Migration direction (**a,c**) and migration speed (**b,d**) are quantified as the direction and speed of the movement of cellular centroid, respectively. The time-series were divided into two parts; the first half and last half of the data were used for estimating the response function and testing it, respectively. The blue and red lines in each panel show observations and reconstructions (before the black dotted vertical line, first half of the data) or predictions (after the black dotted vertical line, last half of the data), respectively. r is the correlation coefficient between the observations and reconstructions/predictions (see Materials and Methods) over the entire time series.

After addressing the roles of Rac1 and Cdc42 in the microscopic behaviors, i.e., the local cellular morphological changes, we lastly focused on the macroscopic behaviors of the migratory cells. Although migratory cells are considered to be polarized such that their molecular composition is different between the leading edge and tail^17^, it is unclear whether the properties of the downstream cascades of Rac1 and Cdc42 are spatially inhomogeneous due to this polarity. To test this, we sought to predict the macroscopic velocity of the cell migration based on the hypothesis that the velocity is directly determined by integrating the microscopic factors that drive the local morphological changes regulated by Rac1 and Cdc42. Surprisingly, the direction and speed of the migration velocity were predicted directly by convoluting the local activities of Rac1 and Cdc42 with the response functions (see Materials and Methods) (Fig. 4a,b for Rac1; Fig. 4c,d for Cdc42). This result supports a “tug-of-war” model in which the downstream cascades of Rac1 and Cdc42 homogeneously exist irrespective of the cell’s polarity and the distributed local driving factors sum to regulate the macroscopic migration behavior (see Discussion).

## Discussion

We proposed a predictive approach to identify how the signals of Rho GTPases are processed in signal transduction during cell migration by combination of image processing and statistical analysis. We clarified that the ‘microscopic’ membrane protrusions and retractions of migratory cells are well predicted by the activity of Rho GTPases. We also found that the response function endowed with the properties of a differentiator can explain the counterintuitive observation that the membrane morphological changes precede the activities of the Rho GTPases. By analyzing the relationship between such predictability and the migration modes, we found that Rac1 and Cdc42 contributed to persistent and random migrations, respectively. Additionally, we proposed a tug-of-war model in which macroscopic cellular behaviors such as migration direction and speed are determined by the integration of the distributed local driving factors based on signaling molecules.

**Figure 5:**
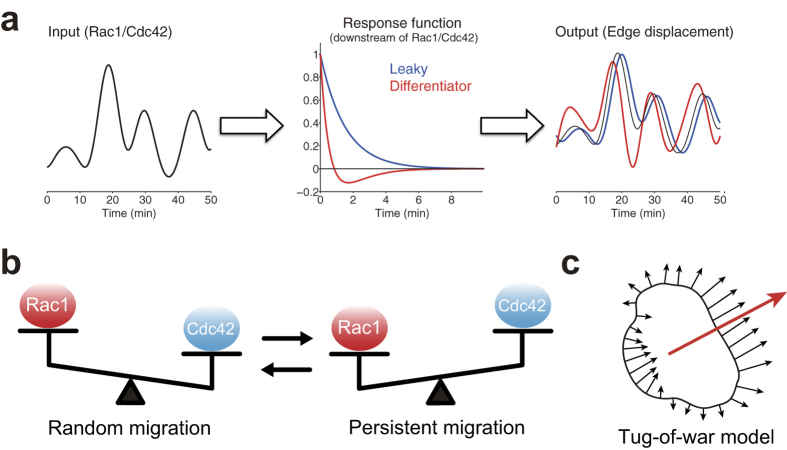
Models of cell migration. (**a**) A model of signal transmission from Rho GTPases to edge displacement in order to explain Rho GTPases activation following membrane protrusion. The response function (middle panel) processes an input signal (Rac1/Cdc42 in the left panel) and generates an output (edge displacement in the right panel). The red and blue curves in the middle panel correspond to the response functions with leaky and differentiating properties, respectively. Note that the estimated response functions possess the differentiating property (right inset in Fig. 2d). In the right panel, output signal through the response function with differentiating property indicated by the red line is followed by input signal, which is plotted by the thin black curve for comparison. (**b**) A model of migration modes depending on the balance between the activities of Rac1 and Cdc42. In persistently and randomly migrating cells, Rac1 and Cdc42 have great contributions to the cell migration, respectively, which can be evaluated in terms of the predictability measure (Fig. 3c). (**c**) A tug-of-war model of macroscopic cell migration. The angle and speed of the cellular migration indicated by the red vector can be predicted by summation like tug-of-war of the distributed driving factors of the locally extended membrane indicated by the black arrows (Fig. 4). The driving factors are predicted by Rac1/Cdc42 through the response function (Fig. 2a).

In contrast to the common belief that Rac1 and Cdc42 positively regulate membrane protrusion^7^, we and others have previously reported that membrane protrusion (retraction) precedes the activation (inactivation) of Rac1 and Cdc42 by 30–60 seconds^14^,^15^,^26^. The decoding analysis described in this study demonstrated that local Rac1 and Cdc42 activities contain sufficient information to predict local morphological changes; therefore, the Rho GTPases are located at the unique and direct upstream juncture of cellular morphodynamics in those cells. Moreover, we clarified that the response functions work as a differentiator (the right inset in Fig. 2d for Cdc42; Supplementary Fig. S2d for Rac1). Then, we proposed that the counterintuitive observation above is due to a side effect of the signal transmission represented by the response functions; the membrane protrusion is induced by a temporal derivative of the Rac1 and Cdc42 activities, especially in the initial phase of the activity increase (Fig. 5a). On the other hand, the property of differentiator has physiological advantage, because the downstream morphodynamics system could possess sensitivity to the input signal, provided by guidance molecules, while keeping adaption ability; actually motile cells are known to be insensitive to the mean level of guidance molecules but sensitive to the gradient^27^,^28^,^29^,^30^,^31^,^32^,^33^,^34^.

In addition, the counterintuitive observation could possibly be the result of actin-mediated feedback effect on the Rho GTPases^10^,^35^,^36^,^37^; Rho GTPase-induced actin polymerization also activates Rho GTPase. The Rho GTPases would further be activated by the positive feedback following membrane protrusion. It should be noted that our decoding analysis is valid even if such feedback existed because the activities of Rho GTPases we used for the decoder could be viewed as already involving these feedback effects.

Our regression analysis of the Cdc42 and Rac1 activities demonstrated that the response functions spatiotemporally exhibited V-shaped profiles (Fig. 2d and Supplementary Fig. S2d); i.e., the local and transient activation of Rac1 and Cdc42 induced membrane protrusion locally but also induced membrane retraction in other perimeters, which could be explained through volume conservation. Another important finding was that the local protrusion propagated laterally. What is the physiological mechanism of this V-shaped response function that induces the elongation of the cellular edge? It has been reported that actin polymerization is locally excited and spatially propagates^38^,^39^,^40^. Machacek *et al*. also reported V-shaped edge elongation in motile cells^16^; according to their model, active Rac1 induces actin polymerization and thereby edge elongation, which is followed by retraction caused by the depletion of the globular actin pool. Meanwhile, actin polymerization propagates laterally via a positive feedback signal from the protrusion^10^,^35^,^36^,^37^, hence inducing a V-shaped edge elongation.

The response function we identified reproduced the cellular decoder from Rho GTPases for morphodynamics in such a way that it reflects the kinetics of the downstream pathway^9^. This knowledge assists in identifying the components of the downstream pathway^22^. For example, if we compare the profiles of the response functions between the native condition and a perturbed condition (e.g., constitutive-active or dominant-negative molecules, pharmacological inhibition of molecules and varied extracellular matrix concentrations^41^,^42^), then we can obtain further information on the quantitative manner in which these molecules exert dynamic control over cellular morphology. Although we tried experiments with Rac1 inhibitor (Rac1 inhibitor V)^43^ and Cdc42 inhibitor (ML-141)^44^, these inhibitors did not substantially reduce the activities of Rac1 and Cdc42 in our cell’s case (data not shown). Our approach not only has a large potential to assist in understanding the theoretical aspects of cellular morphodynamics and migration but also provides a systematic method to actively operate motile cells by optical or uncaged control of regulatory molecules.

Through the cross-cellular predictability analysis, we identified two clusters of cells, predictable cells and unpredictable cells (Fig. 3b). Surprisingly, the V-shaped character of the response functions was consistent not only for predictable cells but also for unpredictable cells, the morphodynamics of which were not accurately predicted even using the response function estimated from the data of themselves. The relatively poor predictability of the unpredictable cells implies that another pathway may also be regulating the morphological changes in those cells, and the V-shaped response function suggests the consistent control of Rho GTPases regardless of the recruitment of such a bypass.

We also identified distinct contributions by Rac1 and Cdc42 to the morphological changes depending on the migration mode. The response function of Rac1 accurately predicted persistently migrating cells, whereas the response function of Cdc42 accurately predicted randomly migrating cells (Fig. 3c). Consistent with our finding, several studies have reported that inhibition of Rac1 caused loss of the directionality of the cell migration in other cell types such as mouse keratinocyte^45^, human umbilical vein endothelial cell^46^, and *Dictyostelium*^47^. On the other hand, it has also been reported that persistent migration is correlated with the inhibition of Rac1 in fibroblasts^48^, which appears to oppose our finding. Note that their experimental condition was three-dimensional culture, whereas experiments in this study and consistent previous reports^45^,^46^,^47^ were performed in two-dimensional culture. Taken together, our study is the first report to imply that Rac1 and Cdc42 natively function distinctly in the persistent and random migration modes, respectively (Fig. 5b).

In addition to Rac1 and Cdc42, RhoA, another member of Rho GTPases, is also an essential regulator in the cell migration; RhoA regulates actomyosin-based membrane contraction via activation of Rho kinase^49^,^50^. Due to mutual inhibition between RhoA and Rac1, opposite polarized activities of RhoA and Rac1 are established in cell polarization^51^,^52^,^53^, and the polarity promotes persistent migration. Previous study with time-lapse FRET imaging of RhoA reported that low and high activities of RhoA are observed with random and persistent migrations, respectively^54^. Because of mutual inhibition between RhoA and Rac1, high (low) activity of Rac1 can be associated with random (persistent) migration, which is not consistent with the results of our study (Figs 3c and 5b).

It may have been asserted that motile cells are structurally and functionally polarized with a leading edge and a retraction tail and that the cellular migration is governed by this polarity. However, we showed in this study that ‘macroscopic’ cellular migration is an outcome of summing the ‘microscopic’ local morphological changes that are regulated by Rho GTPases; through an analysis assuming non-polarized, homogeneous motility, the migration direction and speed could be predicted by integrating the local molecular activities along the cellular periphery (Fig. 4). This finding indicates that there is no control center (at least not downstream of the Rho GTPases) that determines global cellular behaviors; this evidence supports the “tug-of-war model” in which distributed local driving factors emerge to control the macroscopic migration (Fig. 5c).

In intracellular signal transduction of cell migration, a great deal of signals is received through many types of receptors, adhesion molecules and ion channels^17^. On contrary, those various signals are convergent to three types of Rho GTPases, i.e., Rac1, Cdc42 and RhoA^55^, which were previously thought to have distinct functions to induce different subcellular structures, lamellipodia, filopodia and stress fibers, respectively^7^,^9^. Although these subcellular structures are important for inducing driving traction force of the cell migration, how they are coordinated during the cell migration through spatiotemporal regulation of Rho GTPases is remaining as an open question. This question could be solved by the proposed predictive approach in future with identification of the multi-dimensional response functions of Rho GTPases to coordinated movements of lamellipodia, filopodia and stress fibers.

As demonstrated in this study, a predictive approach to extract hidden information processing mechanisms from imaging data is increasingly necessary in the era of high-throughput biological experiments. We believe that such predictive approach provides powerful and broadly applicable tools to further understanding information processing in various biological systems.

## Materials and Methods

### Cells, Plasmids, and Reagents

HT-1080 cells were purchased from the American Type Culture Collection or the Japan Cell Resource Bank and maintained in Dulbecco’s modified Eagle’s medium (DMEM) (Sigma-Aldrich, St. Louis, MO) supplemented with 10% foetal bovine serum (FBS). The cells were transfected with the plasmid encoding the FRET biosensors, Raichu-Rac1/1011x or Raichu-Cdc42/1054 × 1 with Lipofectamine 2000 reagent according to the manufacturer’s protocol (Invitrogen, San Diego, CA).

### Time-lapse FRET Imaging

FRET imaging of Rac1 or Cdc42 activity in randomly migrating HT-1080 cells was performed as described previously^14^,^56^. Briefly, HT-1080 cells expressing FRET biosensors were suspended using trypsin, plated on collagen-coated 35-mm glass-bottomed dishes, and cultured with Phenol Red-free DMEM/F12 (Invitrogen) containing 10% FBS for approximately 1 hour. Prior to imaging, the culture medium was overlaid with mineral oil to prevent evaporation. The cells were imaged with an inverted microscope (IX81; Olympus, Tokyo, Japan) equipped with an UPLANSAPO 60x oil-immersion objective lens (Olympus), a cooled charge-coupled device (CCD) camera (Cool SNAP-K4; Roper Scientific), a light-emitting diode (LED) illumination system (CoolLED precisExcite, Molecular Devices), an IX2-ZDC laser-based autofocusing system (Olympus) with an MDXY30100T-Meta automatically programmable XY stage (Sigma Koki, Tokyo, Japan). The following filters were used for the dual-emission imaging studies: excitation filters, 435/20 for cyan fluorescent protein (CFP) and FRET (Olympus); dichroic mirrors, XF2034 (Omega); and emission filters, 480AF30 for CFP (Omega) and 535AF26 for FRET (Omega). The exposure time was 200-400 milliseconds for CFP and the FRET images when the binning was set to 4 × 4. After background subtraction, the FRET/CFP ratio images were created with the MetaMorph software (Universal Imaging, West Chester, PA) and were represented in the intensity-modulated display mode. In the intensity-modulated display mode, eight colors, from red to blue, were used to represent the FRET/CFP ratio, with the intensity of each color indicating the average intensity of FRET or CFP.

### Cell edge detection

The cellular edge was extracted from the CFP image. The CFP image was first deblurred by blind image deconvolution^57^, in which original image was estimated by maximum likelihood method without explicit knowledge of the point spread function in the microscopy. The deblurred CFP image was then smoothened using a Savitzky-Golay filter^58^, which made each of the local image patches (5 × 5) smooth with a second-order polynomial smoothing kernel. This preprocessing was beneficial for improving accuracy of subsequent cellular edge detection. The smoothed CFP images were then binarized with a certain threshold, which was tuned so that intracellular and extracellular regions were clearly segregated (Supplementary Fig. S3). Note that because Rac1 and Cdc42 include CAAX motif which is membrane targeting signal, Raichu-Rac1 and Raichu-Cdc42 were localized at membrane both at cellular periphery and cortex (Supplementary Fig. S4). Each pixel in the CFP image was classified into either a high-intensity or low-intensity pixel, which corresponded to the intra- and extra-cellular regions, respectively. The threshold value was set to be common over the space and time-lapses for each cell. The small hole-like extracellular regions surrounded by intracellular regions were filled with the intracellular regions. Finally, the cellular edge was obtained as a closed loop of the intracellular region.

### Virtual marker tracking

To track the cellular edge in the process of cell migration, *N* (*N* was 100 in our analysis) markers were virtually placed along the entire cellular periphery in an image plane from the first time point. Basically, minimal travel paths for the virtual markers were searched with the constraints that the markers always had to be on the cellular edge and that adjacent markers had to be equally spaced. These constraints were effective in avoiding unfavorable crossing among the paths and in consistently tracking the markers over an extended observation period. This tracking of the virtual markers was described using an optimization problem to minimize the following cost function:


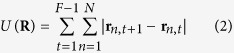


subject to *dist*(**r**_*n,t*_, **r**_*n* ± 1*,t*_; **M**_*t*_) = *L*_*t*_*/N*, where **R** is the set of positional vectors for all of the markers, **r**_*n*,*tn, t*_ = (*x*_*n,t*_, *y*_*n,t*_)^T^ is the 2-dimensional marker position of the *n*-th marker at time *t* (*n* = 1, …, *N*; *t* = 1, …, *T*), *F* is the number of image frames, and *L*_*t*_ is the length of the cellular boundary at time *t*. The |***z***| term denotes the Euclidian norm of vector ***z***. *dist*(**r**_*n,t*_, **r**_*n* ± 1*,t*_; **M**_*t*_) represents the (non-Euclidian) distance between **r**_*n,t*_ and **r**_*n* ± 1*,t*_ along the cellular edge and was calculated based on **M**_*t*_, a set of coordinates of the cellular edge pixels at time *t*.

This constrained optimization problem was solved using the following iterative procedure. First, all of the markers were arbitrarily positioned equidistant from each other in each image frame. In each iteration, the positions of all of the markers in each image frame *t* (**r**_1*,t*_, …, **r**_*N,t*_) were simultaneously updated with the same positional shift along the cellular edge. The shift was calculated based on an average inner product between the gradient of the above cost function with respect to the marker position **r**_*n,t*_ and a normalized tangential vector along the cellular edge:


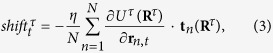


where *η* and *τ* indicate a positive constant and the iteration number of the gradient descent, respectively. The **t**_*n,t*_(**R**^τ^) term indicates the unit tangential vector of the local edge.

### Quantification of Local Membrane Elongation/Retraction

After the marker tracking, the elongation/retraction of the local cellular edge was quantified on each marker between successive image frames. If the Euclidian distance was measured between the positional vectors of the *n*-th marker at time *t* and at time *t + *1, |***r***_*n,t + *1_−***r***_*n,t*_|, it may include not only the membrane elongation/retraction but also the displacement of the cellular centroid due to the migration. To solely quantify the membrane elongation/retraction on the *n*-th marker at time *t*, *d*_*n,t*_, we calculated the orthogonal component of the travelling vector of the *n*-th marker between two time frames *t* and *t* + 1, ***r***_*n,t + *1_−***r***_*n,t*_, to the cellular boundary as follows:


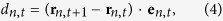


where **e**_*n*,*t*_ denotes the unit normal vector to the local edge within a certain window. The heat map of local morphological changes (Fig. 1c) represents *d_n,t_*.

### Quantification of Rac1 and Cdc42 Activity at the Cell Edge

The activities of Cdc42 and Rac1 at the cell edge were quantified as follows: To obtain the signals of the CFP and FRET channels, a Gaussian convolution kernel was applied to each virtual marker with a standard deviation of 3.8 [pixels] to the images of the CFP and FRET channels. This standard deviation was set as roughly one third of the mean distance between neighboring markers over the set of images. Further, the activities of Cdc42 and Rac1 were obtained as a ratio of the FRET signal to the CFP signal for each virtual marker. Although the Gaussian kernel often convoluted the extracellular pixels, such an unfavorable effect was eliminated when using the ratio.

### Correlation analyses

The spatiotemporal cross- and auto-correlation functions were calculated essentially as described previously^14^,^59^. The cross-correlation *C*_*i,j*_ between *Y*_*n,t*_ and *Z*_*n,t*_ is defined as follows:


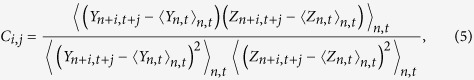


where the operator <  > _*n,t*_ denotes the averages over time and space. *C*_*0,j*_ represents the temporal cross-correlation function. If *Y*_*n,t*_ = *Z*_*n,t*_, *C*_*i,j*_ becomes the auto-correlation.

### Regression Analyses

The regression weights *w*_*i,j*_ (*i* = −*N/2*, …, *N/2*; *j* = −*T*, …, 0) in equation (1) were estimated via ridge regression. The cost function to minimize is given by the following:


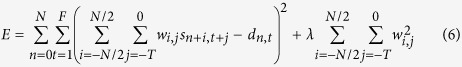


where λ is a positive constant that controls the strength of the regularization term, i.e., the second term in the equation above. In our regression analysis, λ was tuned such to minimize the regression error (the first term in the equation above) in the training dataset.

### Statistical tests

The predictability of the estimated regression model was examined by two statistical tests. First, we checked whether prediction of the edge displacement in validation dataset is improved by using the Rac1 or Cdc42 activity, compared with the simplest prediction in which edge displacement in the validation dataset is predicted as its average in the training dataset. If the prediction was improved by using Rac1 or Cdc42 activity, the variance of the prediction error in the Rac1/Cdc42-based prediction must be significantly smaller than that in the simplest prediction. Then, we confirmed its significance for all predictable cells by the *F*-test (*p* < 0.01 for Cdc42 imaging (N = 7) and *p* < 0.01 for Rac1 imaging (N = 7)).

Second, we also checked whether prediction of the edge displacement in the validation dataset is improved by using history of the Rac1 or Cdc42 activity with the response function, compared with the simple prediction in which the edge displacement is solely predicted by current molecular activity. If the prediction was improved by using history of Rac1 or Cdc42 activity, the correlation coefficient between the predicted and observed edge displacement in the response function-based prediction must be significantly higher than that in the simple prediction. Then, we performed the statistical *t* test after Fisher’s z transformation and confirmed its significance (*p* < 0.01 for all cells in the Cdc42 imaging; *p* < 0.01 for 8 cells and *p* < 0.05 for 2 cells in the Rac1 imaging). These statistical tests for the prediction provided strong evidence that these molecules are responsible for local cellular morphodynamics.

### Similarity between Response Functions

The similarity between the response functions of two cells, *k* and *k’*, was given by cosine similarity defined by the following:


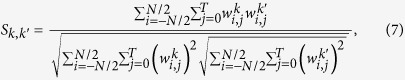


where 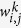 indicates a regression parameter for the spatial distance *i* and delay time *j* estimated from the data of the *k*-th cell.

### Predictions of the Cell Migration Velocity

The macroscopic cell migration, i.e., movement of cellular centroid, is further predicted by integrating the predicted edge-displacements *d*_*n,t*_ (lower panel in Fig. 2a). Under a simplification that the cellular shape is round, the velocity of the cell migration ***V***_*t*_ was calculated as the sum of all of the elongation/retraction markers:


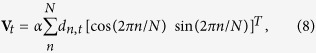


where *α* is a gain constant and was estimated to accommodate to observations. Note that the migration speed and direction were obtained as |*V*_*t*_| and tan^−1^*V*_*t*_, respectively.

## Additional Information

**How to cite this article**: Yamao, M. *et al.* Distinct predictive performance of Rac1 and Cdc42 in cell migration. *Sci. Rep.* **5**, 17527; doi: 10.1038/srep17527 (2015).

## Supplementary Material

Supplementary Information

Supplementary Movie S1

Supplementary Movie S2
