# Supplementary material for: Distinct predictive performance of Rac1 and Cdc42 in cell migration
Source: Sci Rep. 2015 Dec 4;5:17527. doi: 10.1038/srep17527 (PMC4669460; doi:10.1038/srep17527)
Supplement: Supplementary Information [file srep17527-s1.pdf]

Supplementary Information

Distinct predictive performance of Rac1 and Cdc42 in cell migration

Masataka Yamao[1 †], Honda Naoki[2 †], Katsuyuki Kunida[3], Kazuhiro Aoki[2], Michiyuki Matsuda[2, 4], and Shin Ishii[1,2*]

*: Corresponding author

†: These authors contributed equally to this work

1. Graduate School of Informatics, Kyoto University, Sakyo, Kyoto, Japan
2. Imaging Platform for Spatio-temporal Information, Graduate School of Medicine, Kyoto University, Sakyo, Kyoto, Japan
3. Graduate School of Science, University of Tokyo, Bunkyo, Tokyo, Japan
4. Graduate School of Biostudies, Kyoto University, Sakyo, Kyoto, Japan

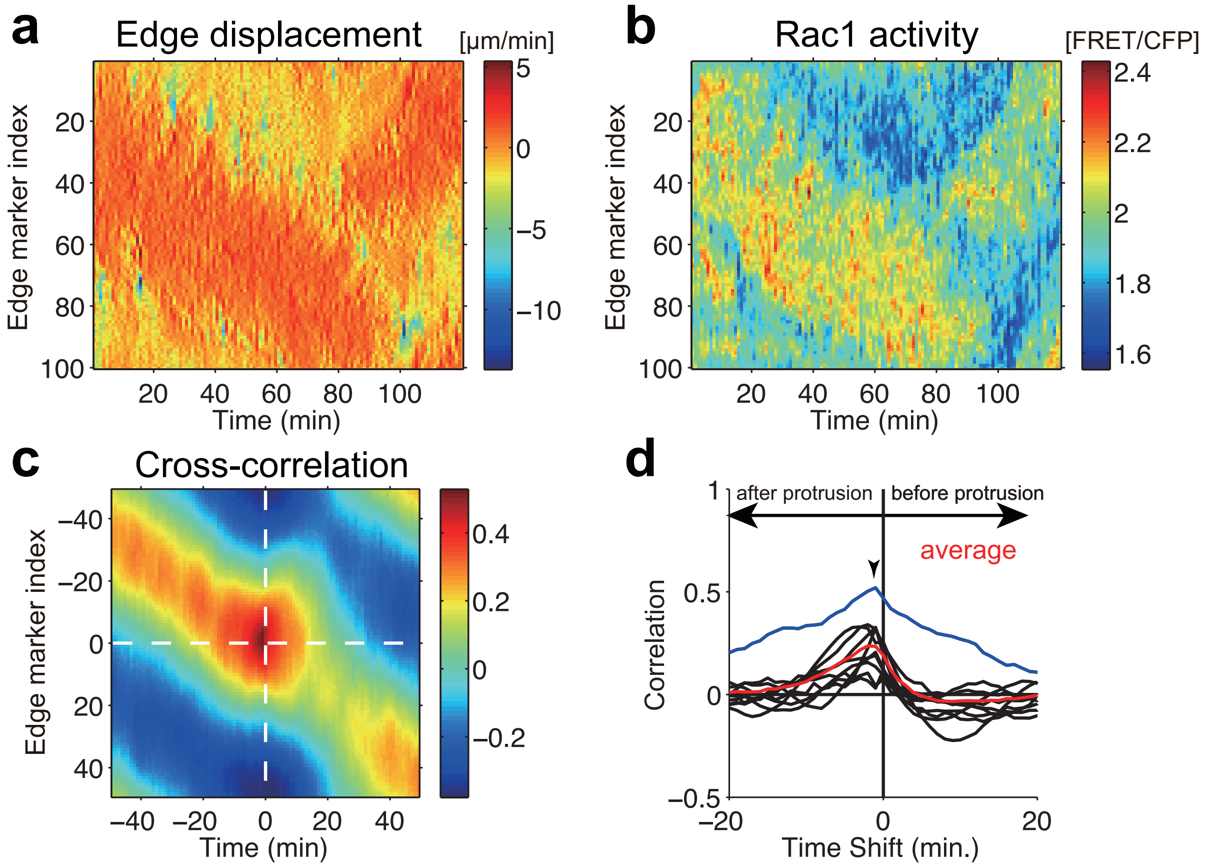

Figure S1. Quantification of cell edge displacement and Rac1 activity

(a, b) Quantified edge displacement (elongation/retraction) **(a)** and Rac1 activity, i.e., the FRET/CFP ratio value **(b)** at each virtual marker is mapped onto a two-dimensional heat map consisting of time (abscissa) and marker index (ordinate).

(c) The spatiotemporal cross-correlation function between the edge displacement and Rac1 activity of a specific cell is plotted. Abscissa and ordinate indicate the shifts in time and marker indices, respectively.

(d) The temporal cross-correlation functions between edge displacement and Rac1 activity are plotted with the time shift. In each sample (a single black line), the local shape change precedes the molecular activity change. The blue line indicates the temporal cross-correlation function of the cell exemplified in **(c)**, i.e., a cross section along the horizontal dotted line in **(c)**. The red line shows the mean cross-correlation function of all of the cells (N = 10).

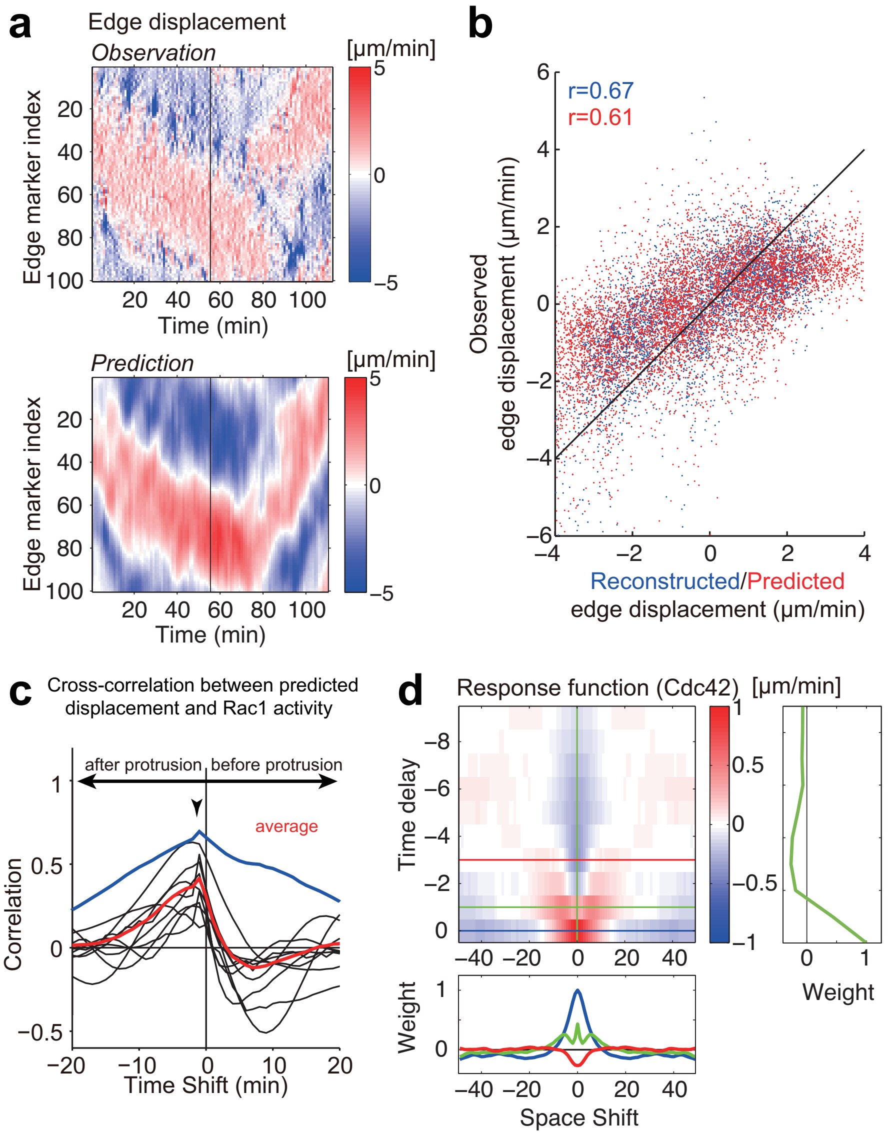

Figure S2. Prediction of elongation/retraction based on Rac1 activity via the response function

(a) Prediction of the local morphological change based on Rac1 activity. The upper and lower panels show the local edge displacement (similar to **Fig. 1c**) obtained in an experiment and the local edge displacement reconstructed/predicted via the response function, respectively. The left-hand side of the vertical black line in the upper panel was used for estimating the response function, and hence the right-hand side was never used for the estimation. For validation, the left-hand and right-hand sides in the lower panel were reconstructed and predicted using the estimated response function, respectively.

(b) The reconstructed and predicted edge displacements were strongly correlated with the observed displacements. Each dot represents the relationship between the reconstruction/prediction and an observation of each edge displacement, and the red and blue colors correspond to reconstruction and prediction, respectively.

(c) The temporal cross-correlation functions between the predicted edge displacement and the Rac1 activity are plotted with the time shift as in **Fig. S1d**.

(d) A response function of Rac1 is plotted on a two-dimensional plane (upper left panel) coordinated by a space shift and a time delay. Each colored-line in the upper right and lower panels represents a cross-section of the spatiotemporal response function (upper left panel) along the straight line with the corresponding color.

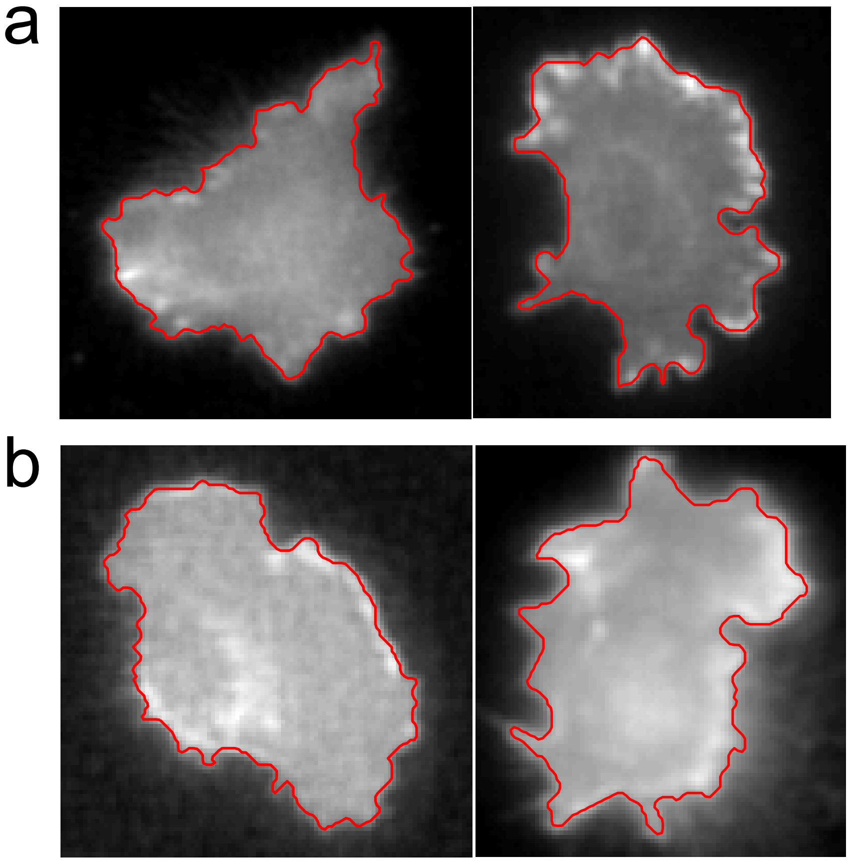

Figure S3. Cellular edge detection

Cellular edges were detected by binarization of the CFP images with an appropriate threshold. Before the binarization, the CFP images were preprocessed by deblurring with blind image deconvolution and smoothing with a Savitzky-Golay filter. Red closed lines indicate the cellular edges detected based on the CFP images of Raichu-Rac1 **(a)** and Raichu-Cdc42 **(b)** in different cells, respectively.

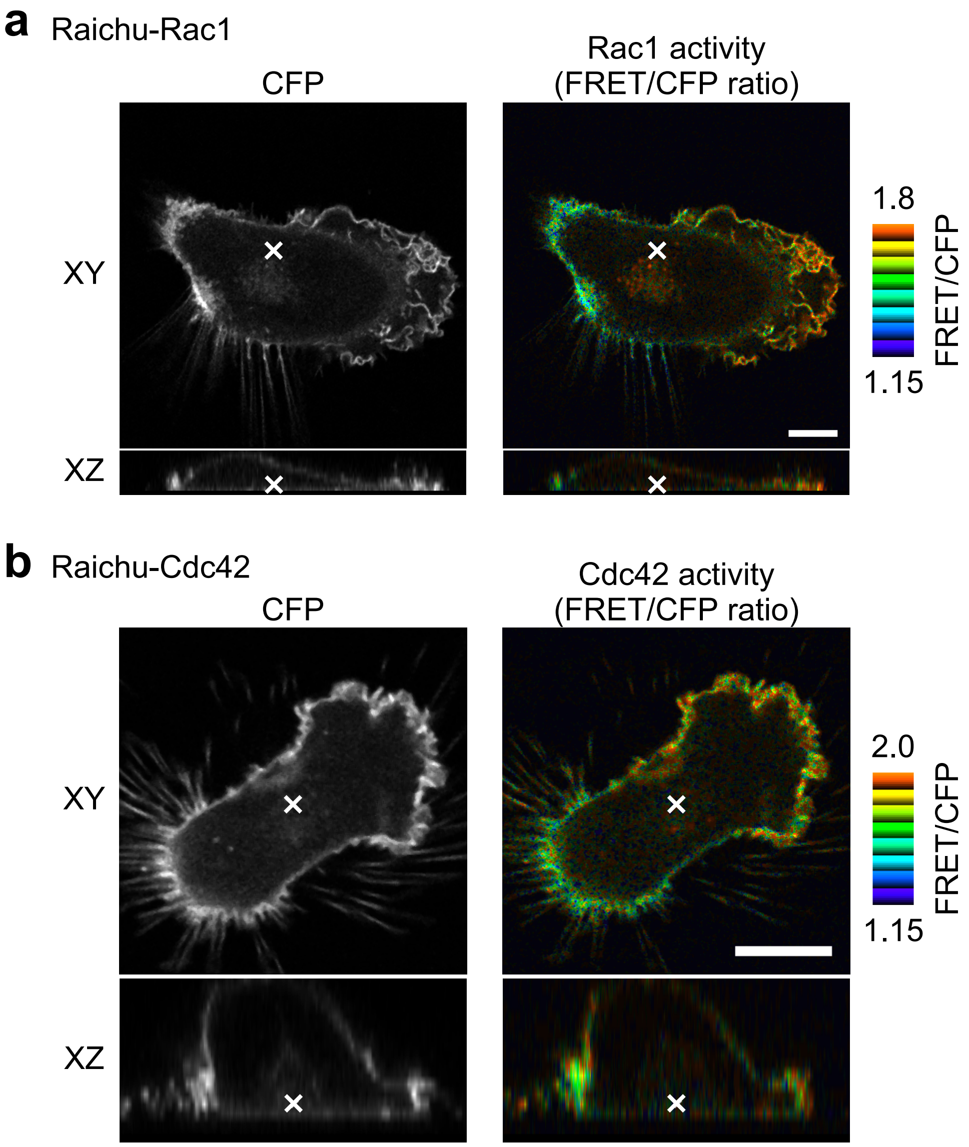

Figure S4. Raichu-Rac1 and Raichu-Cdc42 mainly localize on plasma membrane

Localizations of Raichu-Rac1 **(a)** and Raichu-Cdc42 **(b)** were examined by confocal laser scanning microscopy (see Method of confocal laser scanning microscopy in the Supplementary Information). CFP and FRET/CFP ratio images are represented in left and right panels, respectively. XY and XZ sections are also displayed in upper and lower panels, respectively. A cross line in each panel indicates the position we selected to produce the XZ and XY section images.

Method of confocal laser scanning microscopy

For FRET imaging with a confocal laser scanning microscope, HT-1080 cells were cultured in the same condition as previously reported[1], and imaged using a 60x oil-immersion objective (UPLANSAPO 60X/NA1.35; Olympus Optical Co., Tokyo, Japan) on an IX81/FV1000 inverted microscope (Olympus). The excitation and emission filter settings were as follows: a 440 nm laser diode (Olympus), DM 405–440/515 excitation dichroic mirror, PMT spectral setting 460–500 nm for CFP, and spectral setting 515–615 nm for FRET. Three images were acquired for each plane, and processed with Karman filter to reduce noise. Z-stacks were acquired using 1 μm z-step.

Movie files

Movie S1

HT-1080 cells expressing Raichu-Cdc42 were time-lapse imaged every 1 min by time-lapse microscopy using an inverted microscope (IX81; Olympus) equipped with a x60 objective lens (Olympus). Color represents Cdc42 activity calculated by ratio of FRET images to CFP images; red and blue indicate high and low activities, respectively. Scale bar: 10 μm. Time stamp represents hours:minutes:seconds.

Movie S2

HT-1080 cells expressing Raichu-Rac1 were time-lapse imaged every 1 min by time-lapse microscopy using an inverted microscope (IX81; Olympus, Tokyo, Japan) equipped with a x60 objective lens (Olympus). Color represents Rac1 activity calculated by ratio of FRET images to CFP images; red and blue indicate high and low activities, respectively. Scale bar: 10 μm. Time stamp represents hours:minutes:seconds.

SI References

1 Kunida, K., Matsuda, M. & Aoki, K. FRET imaging and statistical signal processing reveal positive and negative feedback loops regulating the morphology of randomly migrating HT-1080 cells. *J Cell Sci* **125**, 2381-2392 (2012).
